# Sensitive detection of chloroplast movements through changes in leaf cross-polarized reflectance

**DOI:** 10.1093/plphys/kiag739

**Published:** 2026-10-06

**Authors:** Paweł Hermanowicz, Aleksandra Giza, Katarzyna M Sowa, Paweł Korecki, Justyna Łabuz

**Affiliations:** Malopolska Centre of Biotechnology, Jagiellonian University, Gronostajowa 7A, Krakow 30-387, Poland; Malopolska Centre of Biotechnology, Jagiellonian University, Gronostajowa 7A, Krakow 30-387, Poland; Doctoral School of Exact and Natural Sciences, Jagiellonian University, Łojasiewicza 11, Kraków 30-348, Poland; SOLARIS National Synchrotron Radiation Centre, Jagiellonian University, Czerwone Maki 98, Kraków 30-392, Poland; Marian Smoluchowski Institute of Physics, Jagiellonian University, Łojasiewicza 11, Kraków 30-348, Poland; Malopolska Centre of Biotechnology, Jagiellonian University, Gronostajowa 7A, Krakow 30-387, Poland

## Abstract

We show that the positioning of chloroplasts in plant cells affects the ability of photosynthetic organs to depolarize linearly polarized light through diffuse reflection. We apply this phenomenon for the detection of blue-light-induced chloroplast movements using light reflected from leaves and green stems. High-light-induced chloroplast avoidance leads to an increase in reflectance, while chloroplast accumulation in low light has the opposite effect. The changes in reflectance are correlated with an increase in the leaf’s ability to depolarize linearly polarized light. Inclusion of polarization information in the detection procedure allows for the mitigation of the effects of sample orientation and for an increase in the specificity of detection. We have employed this approach to examine chloroplast movements in the model plant *Arabidopsis thaliana* and 4 flowering plant species collected in the field. Despite the importance of chloroplast movements for the optimization of photosynthetic efficiency and biomass production, high-throughput reflectance-based methods are not routinely employed. Our approach opens the possibility of noninvasive, noncontact detection of chloroplast movements in a manner insensitive to the orientation of the leaf.

## Introduction

Chloroplast movements allow plants to achieve high photosynthetic performance and biomass production in dynamic light conditions (Gotoh et al. 2018; Hart et al. 2019). In low light, chloroplasts gather at cell walls perpendicular to the light direction to increase photosynthesis. In high light, chloroplasts move to the walls parallel to the direction of light as a photoprotective mechanism. In flowering plants, chloroplast movements are triggered by blue light, perceived through UV/blue light receptors, phototropins (Banaś et al. 2012; Łabuz et al. 2022). Two phototropins control chloroplast movements in the model plant *Arabidopsis thaliana*. Phot1 and phot2 trigger chloroplast accumulation (Sakai et al. 2001), while sustained chloroplast avoidance is elicited only by phot2 (Jarillo et al. 2001; Kagawa et al. 2001).

Initially, chloroplast movements were examined using light microscopy, as in the pioneering work of Senn (1908). Using modern microscopic techniques, tracking the movement of a single chloroplast within a cell in response to microbeam irradiation is possible (Kagawa and Wada 2000; Tsuboi and Wada 2010; Wada 2013). Fixed leaf cross-sectioning allows for studying chloroplast positioning in multiple mesophyll layers but precludes time-course studies (Higa and Wada 2016). Low throughput of microscopic methods generated interest in approaches based on macroscopic optical properties of leaves. Chloroplast movements cause substantial changes in leaf transmittance and reflectance (Zurzycki 1961). The most widely used approach to detect chloroplast movements consists of the recording of light-induced transmittance changes, both in the laboratory (Inoue and Shibata 1974; Johanssson and Zeidler 2016) and in field conditions (Williams et al. 2003). As red light usually does not induce the movements in flowering plants, it is used to monitor changes in leaf optical properties triggered by actinic blue light. The transmittance-based detection is robust and reproducible, provided that an integrating sphere or the opal method (Shibata 1958; Walczak and Gabrys 1980) is employed to eliminate the dependence of the signal on the sample-detector distance.

The limitation of the transmittance-based method is the need for direct access to the leaf, which reduces its throughput and applicability in field conditions. An alternative approach is to measure changes in leaf reflectance. An increase in reflectance accompanying the avoidance response has been first reported for *Begonia multiflora* (Seybold 1956) and *Lemna trisulca* (Zurzycki 1961). The detection of chloroplast movements through changes in light reflectance has been reported by (Park et al. 1996; Davis et al. 2011; Dutta et al. 2015, 2017; Baránková et al. 2016). Red light leaf reflectance has been used to monitor chloroplast movements in *Arabidopsis* during light treatments in a growth chamber (Dutta et al. 2015). Measurements of hemispherical transmittance and reflectance of leaves of several species revealed that the cell shape substantially affects the ability of chloroplasts to move, influencing blue-light-induced changes in leaf absorptance (Davis et al. 2011). A detailed study of blue-light-induced changes in leaf transmittance and reflectance, performed in *Nicotiana tabacum* (Baránková et al. 2016), demonstrated the effect of refraction at the cell–airspace interface on changes in leaf absorptance due to chloroplast movements. In our previous work (Hermanowicz and Łabuz 2025), we observed an increase in leaf reflectance resulting from the chloroplast avoidance response across the entire visible range, especially in the 580 to 660 nm region. We found that leaves of *Arabidopsis thaliana* and *Nicotiana benthamiana* could be accurately classified according to the chloroplast positioning based on reflectance spectra extracted from hyperspectral images recorded at small incidence and observation angles. Effects of chloroplast positions on the reflectance spectrum can bias estimates of other plant traits based on spectral imagery and vegetation indices (Hermanowicz and Łabuz 2025).

An important drawback of reflectance-based measurements of plant traits is the dependence of the amount of reflected light reaching the detector on the orientation of the leaf (Li et al. 2019). Specular (mirror-like) reflectance exhibits strong angular dependence, hindering chloroplast movement detection, as reported in *Alocasia macrorrhiza* (Gorton et al. 1999). Several potential approaches can be proposed to alleviate the effect of the leaf orientation on the reflectance-based chloroplast movement detection: (ⅰ) simultaneous detection of reflectance at several angles of observation, (ⅱ) measuring the plant or canopy geometry in parallel to reflectance measurements, and (ⅲ) use of polarization-derived information. Angularly resolved reflectance measurements can be taken in the field using goniometric setups at the ground level (Sandmeier et al. 1999; Suomalainen et al. 2009), or remotely using instruments mounted on an airborne platform (Gatebe and King 2016). They allow for thorough characterization of reflection properties of vegetation and were successfully used for detection of multiple plant traits. However, they typically exhibit small throughput and reduced time resolution, due to the need to reposition the detector in ground setups or roll the airborne platform along its axis while circling above the surface under study. As chloroplast movements are a dynamic process, high temporal resolution is important.

Thus, we have examined whether high specificity and accuracy of chloroplast movement detection can be attained by analyzing the polarization of reflected light. The mechanisms of interaction of light with the leaf suggest that even a simple approach, in which unpolarized measuring light is used in combination with a detector equipped with a polarizer, can improve the detection procedure. This is because unpolarized light becomes partially polarized upon specular reflection by the wax layer or the cuticle, with the degree and the plane of polarization dependent on the orientation of the leaf. By contrast, light diffusely reflected from the leaf interior through multiple scattering does not undergo substantial polarization (Vanderbilt et al. 1985). Another approach is to take advantage of the difference in the ability to depolarize light between the diffuse and specular reflection mechanisms. When the leaf is illuminated with a polarized beam, light reflected from the leaf surface usually does not undergo substantial depolarization. By contrast, diffuse reflectance from the leaf interior is considerably depolarized (Rvachev and Guminetskii 1966; Brakke 1994). Multiple scattering is an important mechanism of depolarization (Ghosh and Vitkin 2011). A greater probability of absorption of light due to chloroplast accumulation should increase the extinction of multiply scattered light more than of light scattered only once. This decreases the contribution of multiply scattered light to the reflected flux. The effect of chloroplast avoidance should be opposite. Depolarization of light by the sample gives rise to the cross-polarized reflectance, meaning that the reflected flux contains light of the polarization state perpendicular to that of the incident beam. Thus, we hypothesize that the cross-polarized reflectance contains information about the chloroplast positioning.

To examine the link between chloroplast movements induced by blue actinic light and polarized red light reflectance, we performed measurements in 3 configurations of the experimental setup: (ⅰ) an incident unpolarized beam with one polarizer (analyzer) placed in front of the detector, (ⅱ) an incident polarized beam with an analyzer in front of the detector, and (ⅲ) the same configuration as in (ⅱ), but with the analyzer rotated between the parallel and crossed orientations both before and after blue-light treatment of the leaf. We used the model plant *Arabidopsis thaliana*: wild-type Columbia and photoreceptor mutants, *phot1*, *phot2* (lacking sustained chloroplast avoidance), and *phot1phot2* (disrupted chloroplast movements). We also employed a signaling mutant, *jac1*, deficient in chloroplast accumulation (Suetsugu et al. 2005). These mutants were in the Columbia background, characterized by leaves covered with trichomes. To investigate whether the presence of trichomes affects the performance of the reflectance-based detection of chloroplast movements, we also examined the trichome-less *glabra1* mutant. To evaluate the extent of applicability of the developed methods, we assessed their performance in photosynthetic organs of several wild species.

## Materials and methods

### Plant material

WT Col-0 *Arabidopsis thaliana* (L.) Heynh., *glabra1, phot1* (SALK_088841) (Lehmann et al. 2011), *jac1-3* (WiscDsLox457-460P9) (Hermanowicz et al. 2019), *phot2* (Jarillo et al. 2001), *phot1phot2* (Hermanowicz et al. 2019), were sown in Jiffy-7 pots (Jiffy Products International AS) and stratified at 4 °C for 2 d. Plants were cultivated in a growth chamber (Sanyo MLR 350H) at 23 °C, 80% relative humidity, a photoperiod of 10 h light and 14 h darkness, at photon irradiance of 70 (±15) μmol·m^−2^·s^−1^ (measured at the plane parallel to the rosettes) supplied by fluorescent lamps. The position of plants from different lines in the chamber was randomized, and the trays with plants were rotated 6 to 7 times per week. The experiments were performed on rosette leaves detached from dark-adapted 5-wk-old plants. Leaf chlorophyll content was assessed with the Leaf State Analyzer (Walz, Germany).

Wild plants were collected in March of 2023 and 2024. Plants were harvested in the afternoon, kept dark-adapted in water, and measured the next day. Flowering specimens of *Draba verna* L. (common whitlowgrass) were collected in a xeric meadow covering the south-facing slope of Górka Pychowicka hill (Kraków, 50°1′45″ N 19°53′6″ E). Chloroplast movements were induced by irradiation of the adaxial side of rosette leaves (Fig. 5a)*. Stellaria media* (L.) Vill. (chickweed) was collected in a young birch-alder forest near Jagiellonian University Campus in Kraków, Poland (50°1′44″ N 19°54′6″ E). The adaxial surface of the third leaf from the top of the plant (asterisk in Fig. 5b) was irradiated. *Tussilago farfara* L. (coltsfoot) was collected at human-disturbed sites (lawns and construction sites) near the Jagiellonian University Campus. Chloroplast movements were induced by irradiation of the abaxial (sun-exposed) side of the scales covering flowering shoots (Fig. 6a). *Vaccinium myrtillus* L. (bilberry) stems were collected in a fir forest on the north-facing slope of Mnisko Mt in the Gorce Range, Poland (750 to 800 m a.s.l, 49°34′40″ N 20°2′39″ E). Chloroplast movements were examined in terminal branches (asterisk in Fig. 6b).

### Definitions and conventions

Bidirectional reflectance *R*(*θ_i_*, *φ_i_, θ_v_*, *φ_v_*) (Nicodemus 1965; Schaepman-Strub et al. 2006) is calculated as the ratio between the radiance *L*(*θ*_v_, *φ_v_*) of the sample when viewed along the direction (*θ_v_*, *φ_v_*), and irradiance *E*(*θ_i_*, *φ_i_*) received from the direction (*θ_i_*, *φ_i_*), where *θ* and *φ* are polar and azimuthal angles, respectively.


$$R(\theta_{i},\varphi_{i},\theta_{v},\varphi_{v})=\frac{L(\theta_{v},\varphi_{v})}{E(\theta_{i},\varphi_{i})}$$

In our experiments, the incident beam was collimated to ensure that the direction of incidence was uniform and well defined. For more complex irradiation geometries, a differential definition of bidirectional reflectance should be used. Radiance *L* is an angular quantity, defined as the ratio of power *Φ_d_* (*θ*_v_, *φ_v_*) detected and the product of the angular size *Ω* of the detector with respect to the viewed surface and the area *A* cos (*θ_v_*) of the surface projected in the viewing direction


$$L(\theta_{v},\varphi_{v})=\frac{\Phi_{d}(\theta_{v},\varphi_{v})}{\Omega \;A\;\cos(\theta_{v})\;}$$

When the distance between the detector and the reflecting surface is increased, its angular size decreases with distance squared. The signal produced by the detector also decreases with distance squared, provided that it is far enough from the surface. Thus, radiance, being proportional to the signal/angular size ratio, is the quantity appropriate for characterizing the amount of light reflected in a particular direction by the surface. Irradiance characterizes radiant power $\Phi_{i}(\theta_{i},\;\varphi_{i})$ delivered from the direction $\left(\theta_{i},\;\varphi_{i}\right)$ to the unit area of surface, that is


$$E(\theta_{i},\varphi_{i})=\;\frac{\Phi_{i}(\theta_{i},\varphi_{i})\;}{A}$$

where *A* is the irradiated area. As only radiance is an angular quantity, bidirectional reflectance is expressed in inverse steradians (sr^−1^), or, equivalently, in percents per steradian (%·sr^−1^).

In our experiments, the incident rays, the leaf normal, and the direction of observation were all contained in the same plane, so we specify the directions of incidence and observation using a single signed angle, measured with respect to the leaf normal. A positive angle *θ* corresponds to the polar angle *θ* and azimuth of 0°, while a negative angle −*θ* corresponds to the polar angle *θ* and azimuth of 180° (Fig. 1a). Following (Vanderbilt et al. 1985), we calculated polarized reflectance as the ratio of the polarized radiance and part of the received irradiance corresponding to the appropriate polarization state, even when the incident radiation was unpolarized. In experiments with polarized incident light, total reflectance *ΔR*_tot_ was calculated as the sum of reflectance values obtained for 2 orthogonal orientations of the analyzer (the polarizer in front of the detector). Light polarized in the plane of the normal and the incident ray is referred to as *P*-polarized. Light polarized in the plane perpendicular to the plane of incidence is described as *S*-polarized (Fig. 1b). In our experiments, the extrema of detected signal intensity for light reflected from a leaf coincided with the *P* and *S* orientations. Thus, the degree of linear polarization (DoLP) of reflected light was estimated as


$$\mathrm{DoLP}=\;\frac{R_{P}-R_{S}}{R_{P}+R_{S}}$$

**Figure 1:**
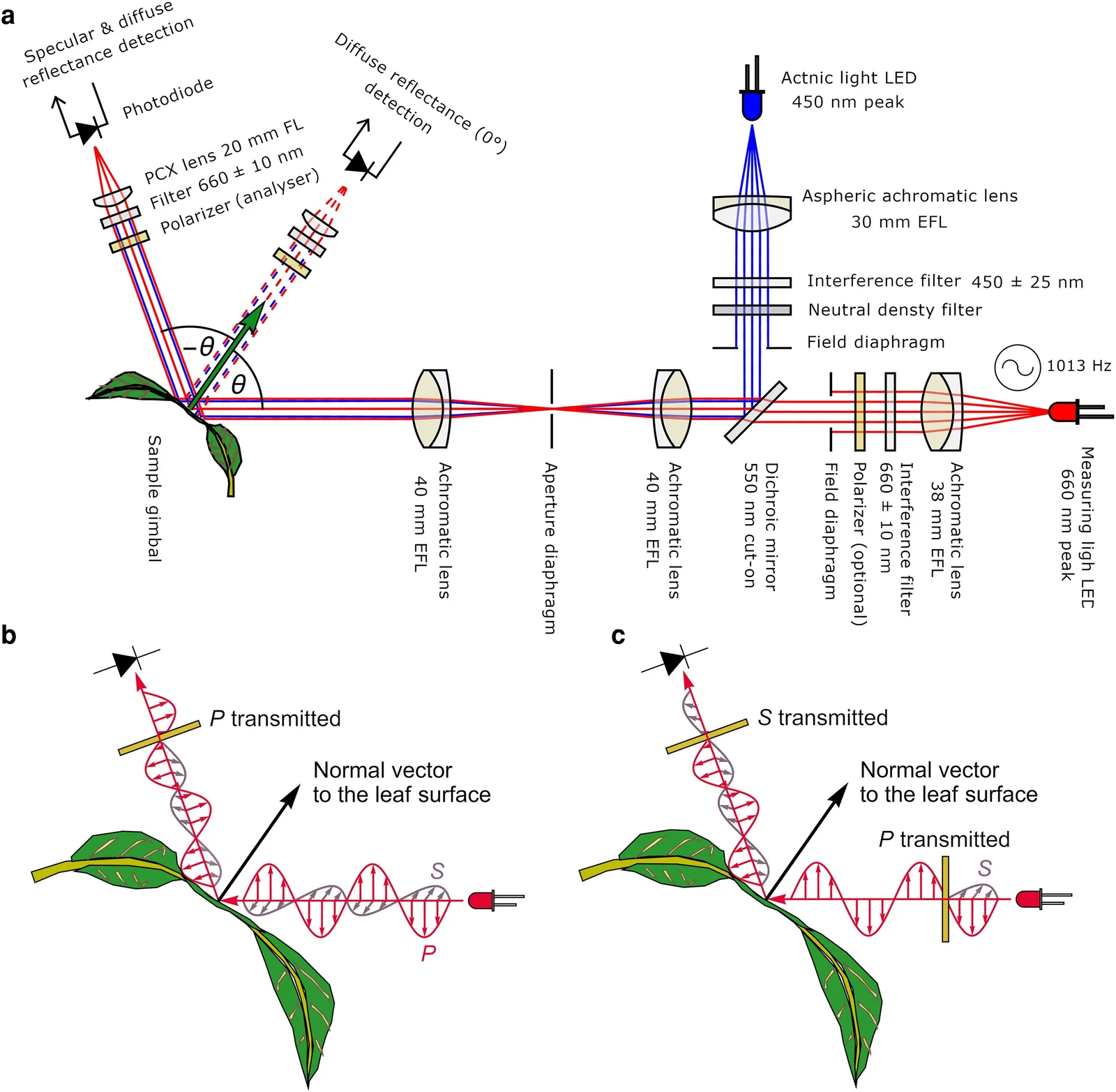
Schematic representation of the reflectometric set-up used in the study. a) Blue (450 nm) and red (660 nm) beams are collimated, combined using a dichroic mirror, and directed towards the leaf. Blue, continuous light induces chloroplast movements. Modulated red light is used to monitor reflectance changes due to chloroplast movements. The angle of incidence *θ* of the combined beams can be changed continuously. The directional reflectance is measured at a variable angle of observation, with the detector placed in the plane spanned by the direction of incident rays and the normal to the leaf. Reflected light is passed through a linear polarizer (analyser) and a filter blocking blue actinic light. It is then focused on the detector. (b, c) Alternative configurations of the set-up. Light polarized in the plane formed by the incident ray and the normal vector to the leaf surface is described as *P*-polarized, while light polarized in the perpendicular direction is *S*-polarized. In b), the incident light was unpolarized, and the reflected light passed through an analyzer in front of the detector that transmitted only one polarization state (*P*-transmitting orientation shown). In c), the incident light was *P-*polarized. The reflected light contained not only *P-*polarized light, but also the cross-polarized (*S*) light due to depolarization by the sample. Only the *S*-polarized component of the reflected light was detected.

where *R_P_* and *R_S_* are reflectance values obtained with the *P* and *S* orientations of the polarizer, respectively.

### Optomechanical setup for measurements of blue light-induced changes in leaf bidirectional reflectance

The changes in red light reflectance of the leaf due to blue-light-induced chloroplast movements were measured with a custom optomechanical setup (Fig. 1a, Figure S1). A detailed description of the setup and calibration procedures (Rowell et al. 1969; Weidner and Hsia 1981; McClain et al. 1995; Newman et al. 2015) can be found in the Supporting Materials 1. Briefly, the actinic beam was produced by a blue LED (455 nm), while the measuring beam was produced by a red LED (660 nm). The red beam was polarized with a linear polarizer in some of our experiments. The beams were collimated and combined with a dichroic mirror, then passed through a condenser and directed towards a leaf mounted in a gimbal. The gimbal allowed for control of the angle between the leaf surface and the direction of incident light. The angle between the incident beam and the leaf normal was kept at 40° or 55°. The angle of 55° corresponds to Brewster's angle for the interface between air and a sample made of material with a refractive index of 1.43, close to the values reported for epicuticular waxes (Jacquemoud and Ustin 2019). When the angle of incidence is equal to Brewster's angle, light reflected specularly from a nonadsorbing, homogeneous, and isotropic material (Meeten 1997) is completely polarized, in the *S* plane, even when the incident beam is unpolarized. In the case of a scattering sample, such as a leaf, light is also reflected diffusely, in all directions, including the direction corresponding to the specular reflection. The reflected light was collected at the observation angles of 0° (diffuse reflection) and either −40° or −55° (diffuse and specular reflection) with respect to the leaf normal. It passed through a linear polarizer (analyzer), a bandpass filter (660 nm) blocking actinic light and most of the chlorophyll fluorescence, and it was finally focused on a photodiode detector. To control the observation angle, the detector was mounted on the rotation stage. The measuring beam was amplitude-modulated at 1013 Hz using a signal lock-in amplifier program (Luda et al. 2019). This approach improves the signal-to-noise ratio and reduces the risk of detection of reflected actinic light. Time courses of leaf reflectance were processed with a custom-written Mathematica (Wolfram Research, USA) script.

### Measurements of blue-light-induced changes in leaf hemispherical transmittance

Hemispherical leaf transmittance changes due to chloroplast movements were assessed with the photometric method (Walczak and Gabrys 1980; Gabryś et al. 2017), using a custom-built photometric setup. The device used the same blue and red LED sources as the reflectometric setup. A detached leaf was mounted in front of a port of an integrating sphere (IS200-4, Thorlabs). The signal was detected with a photodiode detector (DET100A2, Thorlabs), mounted at another port of the sphere. Light reaching the detector was filtered with an absorptive/interference compound filter (660 nm max, BN660, Midwest Optical Systems, USA) to reduce shifts in the transmission band of an interference filter.

### Light measurements

Chloroplast movements were induced with either 0.1 (accumulation) or 100 µmol m^−2^·s^−1^ (avoidance) of blue light. The spectra of actinic and measuring light (Figure S2) used in the reflectometric setup were measured with a BLACK Comet SR spectrometer (25 µm slit, Stellar Net, USA), equipped with a cosine corrector. The irradiance of blue light was measured at the plane of the leaf blade with the LI-190R sensor (LI-COR, USA), combined with the Keithley 6485 picoammeter. Due to the small size of the spot of the red beam at the sample plane in the reflectometric setup, its power was measured with the microscope slide power sensor (S170C, Thorlabs).

### Laser scanning confocal microscopy

Microscopic observations were performed with the Axio Observer.Z1 inverted microscope (Carl Zeiss, Germany) and the LSM 880 confocal module. The long-distance LCI Plan-Apochromat 25×, NA 0.8, objective was used with glycerol immersion. Chlorophyll was excited with the 633 nm He–Ne laser, and fluorescence emission was recorded in the range of 659 to 721 nm. Chloroplast positioning was observed in the parenchyma of plant leaves or stems irradiated in the reflectometer setup for 1 h. Z-stacks were collected on the adaxial surface of *Draba verna* and *Stellaria media* leaves, the abaxial side of *Tussilago farfara* scale leaves, and *Vaccinium myrtillus* stems, with the interval between slices set to ca. 2.4 μm. Projection images were calculated from slices spanning the epidermis and palisade layer (60 to 120 μm of tissue, depending on the species), starting from the upper surface of the epidermis. For cross-sectioning, leaves were fixed with 4% formaldehyde in 50 mM PIPES, 10 mM EGTA, 5 mM MgSO_4_, pH 7.0, and embedded in 6% (w/v) low-melting-point agarose (Agarose Super LM, Carl Roth). Leaf cross-sections of 100 to 150 μm in thickness were prepared using the 7000smz−2 vibrating microtome (Campden Instruments, UK) with a ceramic blade (7550-1-SS, Campden Instruments). The stems of *V. myrtillus* were cut manually with a razor blade.

### X-ray microtomography

For X-ray tomography, freshly collected leaves were cut into small pieces with razor blades and embedded in polyamide (Kapton) tape following a procedure described in (Théroux-Rancourt et al. 2017). X-ray microtomography (µCT) experiments were carried out in parallel beam geometry at the POLYX beamline (Sowa et al. 2023) of the SOLARIS National Synchrotron Radiation Center (Szlachetko et al. 2023). White X-ray beam from SOLARIS bending magnet was filtered using an Al attenuator with a thickness of 0.5 mm and beamline windows (250 µm Be and 150 µm CVD diamond), resulting in a polychromatic X-ray beam with a central energy of approximately 15 keV. Samples on the rotational stage were placed at a distance of 14.5 m from the source. X-ray projections were acquired using a white beam X-ray microscope (Optique Peter, France) with 10 µm or 50 µm thick LuAG:Ce scintillators (Crytur, Czech Republic) and a 10× objective. The sample-to-scintillator distance was set to 40 mm. Images were captured with a sCMOS camera PCO Edge 5.5 with a 6.5 µm pixel size. Tomographic scans were performed over a 180° range by a continuous rotation of the sample. For each sample, 2,001 equiangular X-ray projections were acquired with a single frame exposure time of 540 ms, yielding a total scan time of around 20 min. Raw X-ray projections were corrected with flat and dark frames and post-processed with stripe suppression and automatic alignment procedures. Phase retrieval was performed with the Paganin method (Paganin et al. 2002) and *δ*/*β* of 100. Tomographic reconstruction was performed using Astra Toolbox (Van Aarle et al. 2016) with an effective voxel size of 0.72 µm. Further segmentation and visualization of tomographic data were performed with a custom-written Mathematica (Wolfram Research, USA) script.

### Statistical analysis

The reflectance change distributions substantially deviate from normality when the detector receives specularly reflected light. The distributions are heavy-tailed, with an excess of extreme values compared with the normal distribution (ie, high kurtosis). The specular lobe of the leaf reflectance function is sharp, so even relatively small changes in leaf undulations during measurement (*eg*, induced by the actinic light) may cause part of the specularly reflected beam to miss the detector. In addition, when specularly reflected light is collected, the observed variance depends on the orientation of the polarizer; thus, the standard homoskedasticity assumption of ANOVA is not satisfied. To reduce the effect of extreme observations, we have transformed the amplitudes and rates of reflectance changes using the inverse hyperbolic sine transformation of the form $\sin\;h^{-1}(\theta x)/\theta$, where *x* is the untransformed value of the response variable, and *θ* is a parameter that needs to be estimated. The linear model of the effect of the plant line, polarizer orientation, and their interaction on the transformed change of reflectance due to actinic light was fitted using the maximum log-likelihood estimator in the *nlme* package (Pinheiro et al. 2023) of the R software (R Core Team 2019). The model allowed for heteroskedasticity, with the variance free to vary with the orientation of the polarizers. The parameter *θ* of the transformation was found through maximization of the concentrated log-likelihood (Burbidge et al. 1988), calculated as the sum of the logarithm of the determinant of the Jacobian of the transformation and the maximum log-likelihood for the linear fit. The values of *θ* that gave the greatest concentrated log-likelihood were selected. The significance of differences in means of transformed values between groups was assessed with Tukey's method, using the *emmeans* (Lenth 2023) and *multcomp* packages (Hothorn et al. 2008). The amplitudes and rates of hemispherical transmittance changes followed the normal distribution, so the statistical analysis was performed on untransformed values. The statistical analysis of transmittance changes was otherwise performed in the same way as for the reflectance measurements. In all figures, each dot represents a value obtained for one leaf, with a single leaf harvested from each plant. Boxes show interquartile ranges, while the horizontal bars mark medians.

## Results

### Chloroplast movements determined by leaf reflectance using unpolarized incident light and polarization-sensitive detection

In this variant of the measurements, only a polarizer in front of the detector (analyser) was used (the incident light was unpolarized, Fig. 1b). To assess the effect of *Arabidopsis* leaf orientation on reflectance and its ability to polarize incident unpolarized red light, we measured the power of the *P-* and *S-*polarized components of light reflected from dark-adapted leaves at 2 angles of incidence and multiple observation angles (Fig. 2a and b). In the *Arabidopsis* wild-type and all mutant lines (*phot1*, *phot2*, *phot1phot2*, *jac1*, and *glabra1*), the average bidirectional reflectance (*R_P_*  *+*  *R_s_*)/2 strongly depended on the observation angle (Fig. 2a, b, and  Figure S3). For wild-type leaves, the average reflectance in the direction of specular reflection was greater than the average reflectance in the direction normal to the leaf surface (Fig. 2a, b). A similar dependence of the bidirectional reflectance on the observation angle was observed for other *Arabidopsis* lines (Figure S3). Interestingly, the reflected radiance was the largest for viewing angles exceeding the angle corresponding to the specular reflection from a flat surface. This counterintuitive phenomenon has been previously observed in leaves of several species. It can be explained by Fresnel's equations of reflectance when the leaf surface is modeled as rough at the microscopic scale, being composed of facets with randomly distributed normal vectors (Torrance and Sparrow 1967; Bousquet et al. 2005).

**Figure 2:**
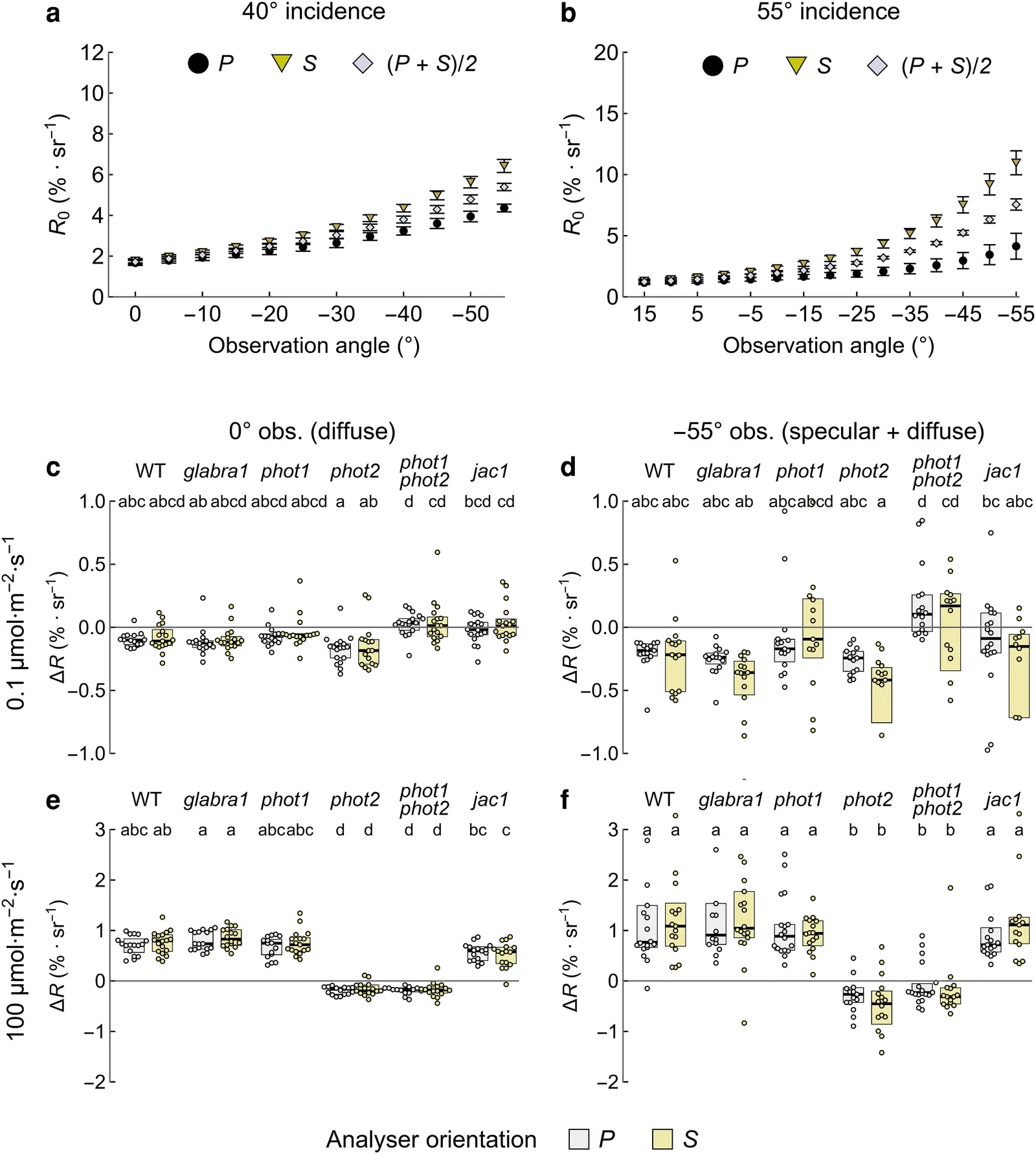
Red-light reflectance of *Arabidopsis* leaves. (a, b) The reflectance was measured for the adaxial side of dark-adapted wild-type leaves, at the incidence angle of 40° a) or 55° b), and at several angles of observation. The incident beam was unpolarized. Either the *P-* (black dots) or *S-* (yellow triangles) polarized component of reflected light was detected. Five leaves from different plants were examined for each combination of plant line and incidence angle. Error bars show the standard error. (c–f) Changes in leaf reflectance due to chloroplast movements. The amplitudes of red-light reflectance changes induced by 1 h irradiation with blue light of 0.1 (c, d) or 100 µmol·m^−2^·s^−1^ (e, f) in leaves of wild-type (WT), *glabra1,* and chloroplast movement mutants (*phot1*, *phot2*, *phot1phot2*, *jac1*). The blue actinic and red measuring beams were unpolarized and impinged on the adaxial leaf surface at 55°. The observation angle was 0° (c, e, diffuse directional reflectance recorded) or −55° (d, f, combined specular and diffuse directional reflectance recorded). Either *P*- (gray boxes on the left for each line, specular reflection attenuated) or *S*- (yellow boxes on the right for each line) polarized component of reflected light was detected. Boxes that do not share any letter represent groups for which the means of transformed values differ at the 0.05 level (Tukey's method, adjusted for multiple comparisons).

To test the accuracy of the reflectance-based monitoring of chloroplast movements, we performed reflectance and transmittance detection in parallel on the same sets of plants. In low blue light of 0.1 µmol·m^−2^·s^−1^, the accumulation response observed in wild-type, *glabra1, phot1,* and *phot2* leaves resulted in a substantial decrease in directional reflectance (40° incidence in Figures S4a, b, S5; and 55° incidence in Fig. 2c, d, Figure S6) and transmittance (Figure S7a, C). In the *phot1phot2* mutant, which lacks both phototropins, the changes in transmittance and reflectance were absent. This indicates that the differences observed in wild-type plants were phototropin-dependent. In the high blue light of 100 µmol·m^−2^·s^−1^, the avoidance response observed in wild type, *glabra1*, *phot1,* and *jac1* leaves resulted in an increase in reflectance at both 40° (Figures S4 c, d and S8) and 55° (Fig. 2e, f, Figure S9) incidence angles and in transmittance (Figure S7b, c). The accumulation response observed in *phot2* had the opposite effect. In most cases, no substantial differences in the magnitude of reflectance changes were observed between leaves of wild-type Columbia and those of the *glabra1* mutant (Fig. 2c–f and Figure S4a–d), which lack trichomes on their surface. The rates of the changes in leaf reflectance and transmittance are shown in Figures S7d and S10.

The changes in the power of the *P-*polarized component of reflected light were similar in magnitude to changes in the power of the *S-*polarized component, and their means were not statistically different. This was observed for both low (Figure S4a, b, and Fig. 2c, d) and high light (Figure S4c, d, and Fig. 2e,f) treatments in all *Arabidopsis* lines. The variance in the reflectance changes was smaller for the *P* component than for the S-component, indicating that specular reflection increases measurement uncertainty. As expected, the initial reflectance *R_0_* of dark-adapted leaves was substantially greater for *S*- than *P*-polarized light (Figure S11). As in (Carter 1991; Baránková et al. 2016), we defined the differential sensitivity of detection as Δ*R*/*R*_0_ (Figure S12). Such sensitivity was substantially affected by the polarizer orientation when specularly reflected light reached the detector. At Brewster's angle (55°), the sensitivity of detection for chloroplast avoidance in the wild type was almost 3 times greater when the polarizer was in the *P* orientation as opposed to the *S* orientation (Figure S12h). The absence of any substantial effect of the polarizer orientation on raw values of Δ*R* was consistent with the chloroplast positioning affecting only the diffuse reflectance component, originating from the mesophyll through multiple scattering (Fig. 2).

### Effect of chloroplast movements on the cross-polarized reflectance

To further reduce the specular component and improve the detection sensitivity of chloroplast movements, we performed experiments with a linearly polarized incident measuring beam (Fig. 1c, Fig. 3). We examined the effects of chloroplast movements induced by high blue light on reflectance for 4 combinations of the polarizer-analyzer orientations: *P*(incident)/*P*(observed); *P*/*S*; *S*/*P*; *S*/*S*. The experiments were performed on the *Arabidopsis* wild-type and the *phot1phot2* mutant. The angle of incidence was set to 55°, while the angle of observation was either 0° (diffusely reflected light recorded) or −55° (specularly and diffusely reflected light). The sensitivity of detection, Δ*R*/*R*_0_, of the avoidance response in wild-type plants was the highest when the polarizers were crossed, ie, for the *P*/*S* and *S*/*P* combinations. The cross-polarized diffuse reflectance (0°observation) increased 2 times after irradiation (Fig. 3a, e). Consistently, this effect was absent in the *phot1phot2* mutant, which lacks chloroplast movements (Fig. 3c, e). The small Δ*R*/*R*_0_ values observed when the detector collected specular reflection and both the incident and observed light were *S*-polarized (Fig. 3b, f) were due to the high levels of initial reflectance *R*_0_ (Fig. 3h), as implied by the Fresnel equations. The measured changes in reflectance and the rates are shown in Figure S13.

**Figure 3:**
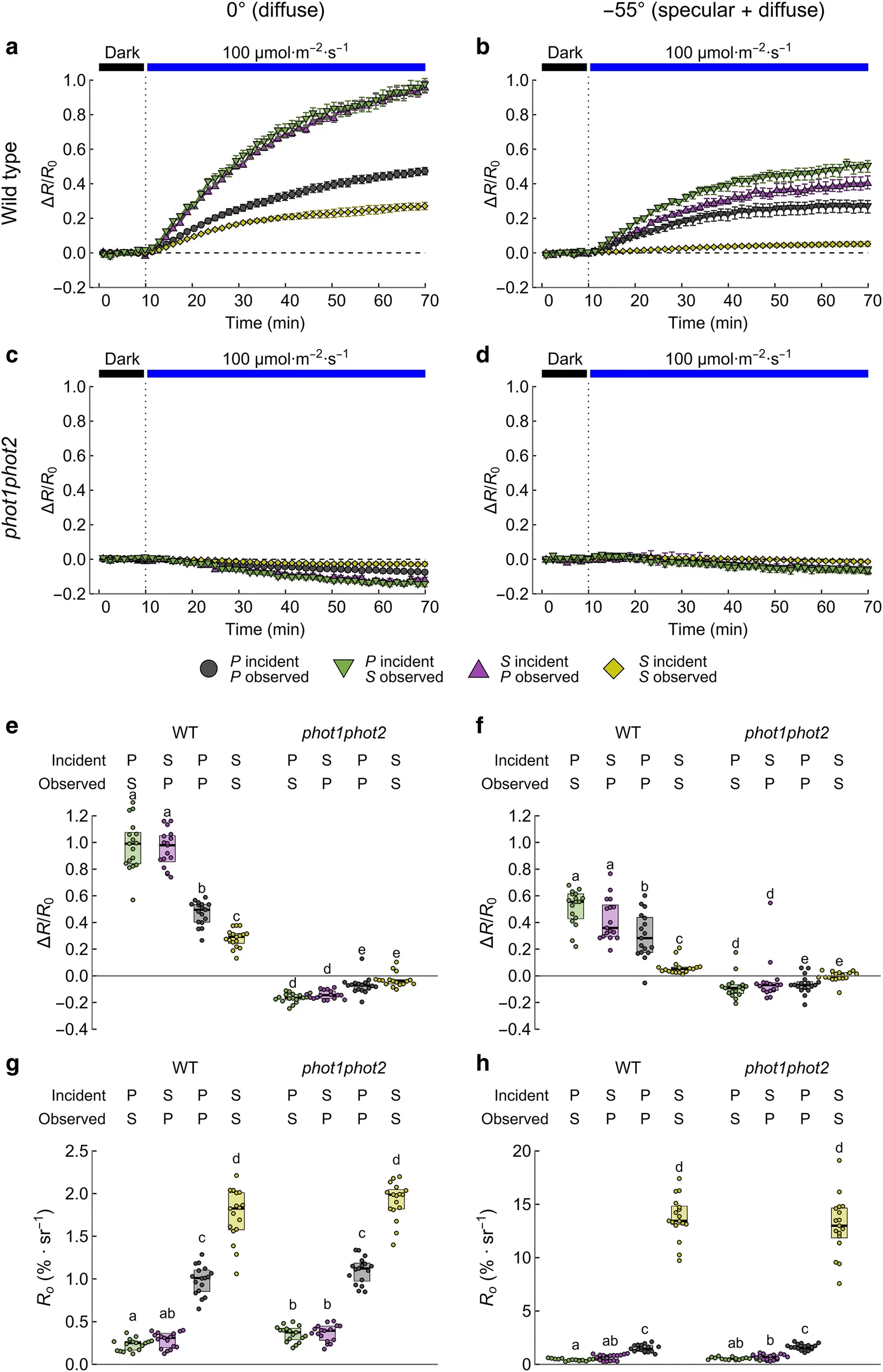
Sensitivity of reflectance-based chloroplast movement detection for different polarizer orientations. (a–d) Time courses of normalized changes *ΔR/R_0_* in the polarized red-light reflectance during 1 h irradiation with high blue light (100 µmol·m^−2^·s^−1^) of the adaxial surface of leaves of wild-type (WT) (a, b) and *phot1phot2* (c, d) *Arabidopsis* plants. The time courses were used to calculate normalized amplitudes shown in (e, f). Initial reflectance *R*_0_ of dark-adapted leaves (g, h) was used for normalization. The incidence angle of blue actinic and red measuring beams was 55°. For measurements of diffuse reflectance (a, c, e, g), the observation angle was 0°. For measurements of combined specular and diffuse reflectance (b, d, f, h), the observation angle was −55°. Four possible combinations of the orientation (*P/S*) of the polarizers in front of the measuring light source and the detector were examined. Boxes that do not share any letter represent groups for which the means of transformed values differ at the 0.05 level (Tukey's method, adjusted for multiple comparisons).

### The relation between the chloroplast positioning and the leaf’s ability to depolarize light

The observed increase in the cross-polarized reflectance may stem from an increase in the leaf’s ability to depolarize linearly polarized light, associated with chloroplast avoidance. To test this hypothesis, we examined how chloroplast movements affect the DoLP of the beam reflected from leaves irradiated with red *P-*polarized light. We performed the measurements for the adaxial (upper) and abaxial (lower) sides of wild-type and *phot1phot2 Arabidopsis* leaves. We measured the power of *P-* and *S*-components of reflected light at 55° incidence and 0° observation angles, before and after irradiation with low (Figure S14a–d) or high blue light (Figure S14e–h). Additional measurements were taken for the adaxial side at the observation angle of −55°, ie, in the specular direction (Figure S15).

For the wild-type *Arabidopsis* plants, DoLP of red light reflected from a leaf irradiated with low blue light was greater than DoLP of red light reflected from the leaf in its dark-adapted state (ΔDoLP > 0) (Fig. 4a). This effect was absent in the *phot1phot2* mutant. By contrast, the degree of polarization of red light reflected from the wild-type leaves decreased after irradiation with high blue light (ΔDoLP < 0) (Fig. 4b). Irradiation of the adaxial side of *phot1phot2* leaves with high blue light had the opposite effect to that observed for the wild type. An increase in DoLP of the reflected red beam was observed. For the dark-adapted leaves of both wild-type and *phot1phot2*, the light reflected from the adaxial side retained a greater degree of polarization than the light reflected from the abaxial side (Figure S16a). Leaf's ability to depolarize light was correlated with its total reflectance (Fig. 4c, d). In wild-type plants, *ΔR*_tot_ decreased after irradiation with low blue light and increased after irradiation with high blue light, regardless of the leaf side. This effect was not observed for the *phot1phot2* mutant, indicating that it results directly from chloroplast movements (Fig. 4e, f). Measurements of the hemispherical transmittance changes were taken in parallel as a control, for both leaf sides (Figure S16c, d).

**Figure 4:**
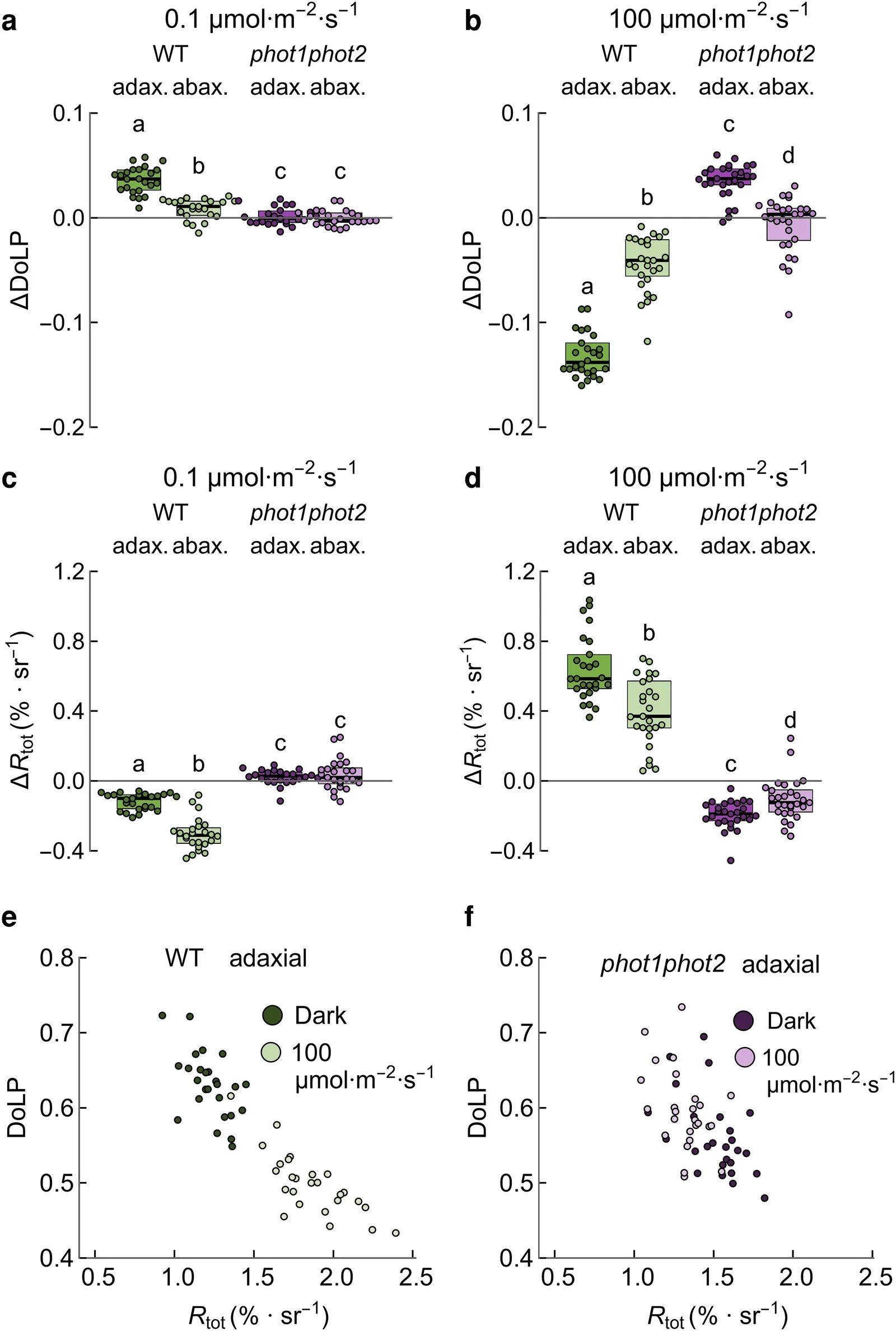
Effect of chloroplast movements on leaf's ability to depolarize linearly polarized light. Adaxial or abaxial surfaces of wild-type (WT) and *phot1phot2 Arabidopsis* leaves were irradiated for 1 h with blue light of low (0.1) or high (100 µmol·m^−2^·s^−1^) irradiance. The measuring red light was *P*-polarized, incident at 55°. The observation angle was 0°. (a–b) Changes in the DoLP of red light reflected from the leaves after irradiation with low a) or high (b) blue light. (c–d) The total red-light reflectance changes after low c) or high d) light. (e–f) The relationship between the total reflectance and the degree of polarization of reflected red light from leaves that were dark-adapted or irradiated with high blue light, for WT e) and the *phot1phot2* line f). Boxes that do not share any letter represent groups for which the means of transformed values differ at the 0.05 level (Tukey's method, adjusted for multiple comparisons).

### Anatomical and physiological factors affecting cross-polarized reflectance

The ability of several materials to depolarize light has been previously shown to increase with their porosity (Egan et al. 1968). The upper (adaxial) part of the mesophyll in leaves of dicotyledonous plants has a compact form of the palisade parenchyma, containing small intercellular spaces. By contrast, the lower (abaxial) part of the mesophyll is occupied by the spongy parenchyma, characterized by high porosity due to large, air-filled spaces (Ma et al. 1990; Mathers et al. 2018). Differences in the porosity of both types of parenchyma were visible in the confocal (Figure S17a) and X-ray microtomography images (Figure S17b) of *Arabidopsis* leaves. Light reflected from the abaxial side was more depolarized than light reflected from the adaxial side.

Both experimental results and radiative transfer models describing the depolarization of light in a scattering medium (Ma et al. 1990; Kalshoven et al. 1995) suggest that high chlorophyll content may reduce the leaf's ability to depolarize light. Wild-type *Arabidopsis* leaves exhibited higher total chlorophyll content than leaves of the phototropin double mutant (Figure S16b). In line with this, light reflected from the adaxial side of wild-type leaves retained a high DoLP as compared to *phot1phot2* leaves (Figure S16a).

We compared the sensitivity of detection based on cross-polarized reflectance (*P*-incident, *S*-analyzed, 0° observation angle) of both accumulation and avoidance responses with that of the transmittance-based detection (Figure S18). To examine nonspecific changes in the leaf reflectance, which may be induced, *eg*, by the measuring light or leaf dehydration, we also assessed the effects of the red probe light applied without the actinic blue light. In *Arabidopsis*, both changes in cross-polarized reflectance to low and high blue light were easily detected (Figure S18a, b). No substantial difference was observed when the measuring light was applied without the actinic beam, indicating that the effect of wilting was negligible. The ratio Δ*R*/*R*_0_ and the analogous sensitivity calculated for hemispherical transmittance, Δ*T*/*T*_0_ (Figure S18c, d), were comparable in magnitude, and the concomitant changes in chloroplast positions were confirmed using microscopic observations explicitly (Figure S18e). Changes in reflectance and transmittance in response to high light differed in their kinetics. Transmittance reached a clear plateau after ca. 30 min of blue-light irradiation. By contrast, reflectance continued to grow throughout the 1-h irradiation phase.

The adaxial and abaxial sides of leaves in dicots usually differ substantially in their reflectance. We examined the effect of leaf side on cross-polarized reflectance changes in *Arabidopsis*, observed at the angle of 0°, induced by 1 h irradiation with high (100 µmol m^−2^ s^−1^) blue light. The increase in cross-polarized reflectance in wild-type plants was similar for the adaxial and abaxial sides (Figure S19a, d), even though the initial reflectance *R*_0_ of the abaxial side was ca. 4 times greater (Figure S19c). Interestingly, greater maximum rate of reflectance due to chloroplast movements was observed for the abaxial side than the adaxial side (Figure S19b).

### Detection of chloroplast movements using cross-polarized reflectance in species collected in the natural environment

To explore the applicability of cross-polarized reflectance for the detection of chloroplast movements, we examined detached photosynthetic organs of 4 wild species, irradiated with 0 (measuring beam only), 0.1, or 100 µmol·m^−2^·s^−1^ of blue light. The measurements were recorded on rosette leaves of *Draba verna* (Fig. 5a), a close relative of *Arabidopsis thaliana*, cauline leaves of *Stellaria media* (Fig. 5b), scales covering flowering shoots of *Tussilago farfara* (Fig. 6a), and green stems of *Vaccinium myrtillus* (Fig. 6b). The red measuring beam did not induce substantial changes in reflectance or transmittance in any of the investigated species. Pronounced chloroplast accumulation and avoidance responses were observed through microscopic observations in *D. verna* (Fig. 5a) and *S. media* (Fig. 5b) leaves. As in *Arabidopsis*, the accumulation response resulted in a substantial decrease in cross-polarized reflectance and transmittance in both species, whereas the avoidance response had the opposite effect. In the scales of *T. farfara*, only the avoidance response to high blue light could be observed microscopically. It was accompanied by an easily detectable increase in the reflectance and transmittance (Fig. 6a). A small but statistically significant decrease in the transmittance and reflectance, corresponding to the chloroplast accumulation, was observed after low blue light irradiation. We also investigated the chloroplast movements in green, photosynthetic stems of *Vaccinium myrtillus,* collected in early spring, at the beginning of bud break (Fig. 6b). While the transmittance-based method could not be used for opaque stems, the sizeable changes in cross-polarized reflectance induced by irradiation with high blue light pointed to the chloroplast avoidance response. Low blue light led to a decrease in reflectance, indicative of chloroplast accumulation.

**Figure 5:**
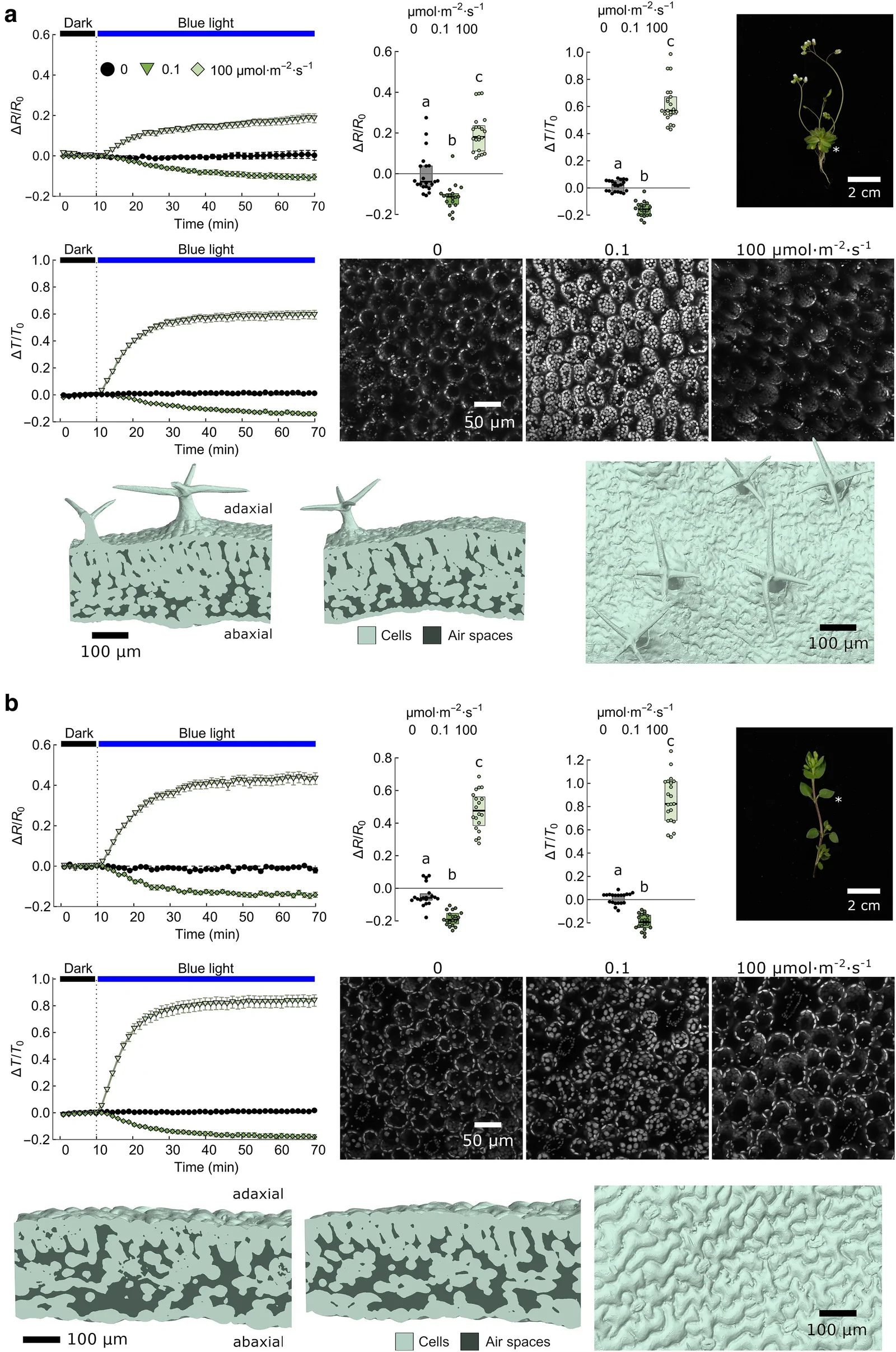
Detection of chloroplast movements by cross-polarized reflectance changes in leaves of wild species. Chloroplast movements were examined through measurements of reflectance and transmittance, as well as microscopic observations, for *Draba verna* a) and *Stellaria media* b) leaves. Time courses show changes in red-light reflectance Δ*R* induced by 0.1 or 100 µmol·m^−2^·s^−1^ blue-light or mock irradiation of the adaxial side of leaves, normalized by the reflectance $R_{0}$ of the leaf in a dark-adapted state. The angle of incidence of beams was 55° while the angle of observation was 0° (diffuse reflectance recorded). The incident red light was *P*-polarized, while the *S*-polarized component of reflected light was measured. Changes in hemispherical transmittance Δ*T* from photometric measurements are shown for reference. Confocal microscopy images of the palisade mesophyll were recorded after 1 h irradiation with blue light of 0.1, 100 µmol·m^−2^·s^−1^ or mock irradiation. Chlorophyll autofluorescence was detected. Photographs show the developmental stage of plants at the time of the measurement, with the examined leaves marked with asterisks. The microtomography images of *Draba verna* a), *Stellaria media* b) show the leaf cross-section and its adaxial surface. In box plots, the means of transformed values from groups that do not share any letter differ at the 0.05 level (Tukey's method, adjusted for multiple comparisons).

**Figure 6:**
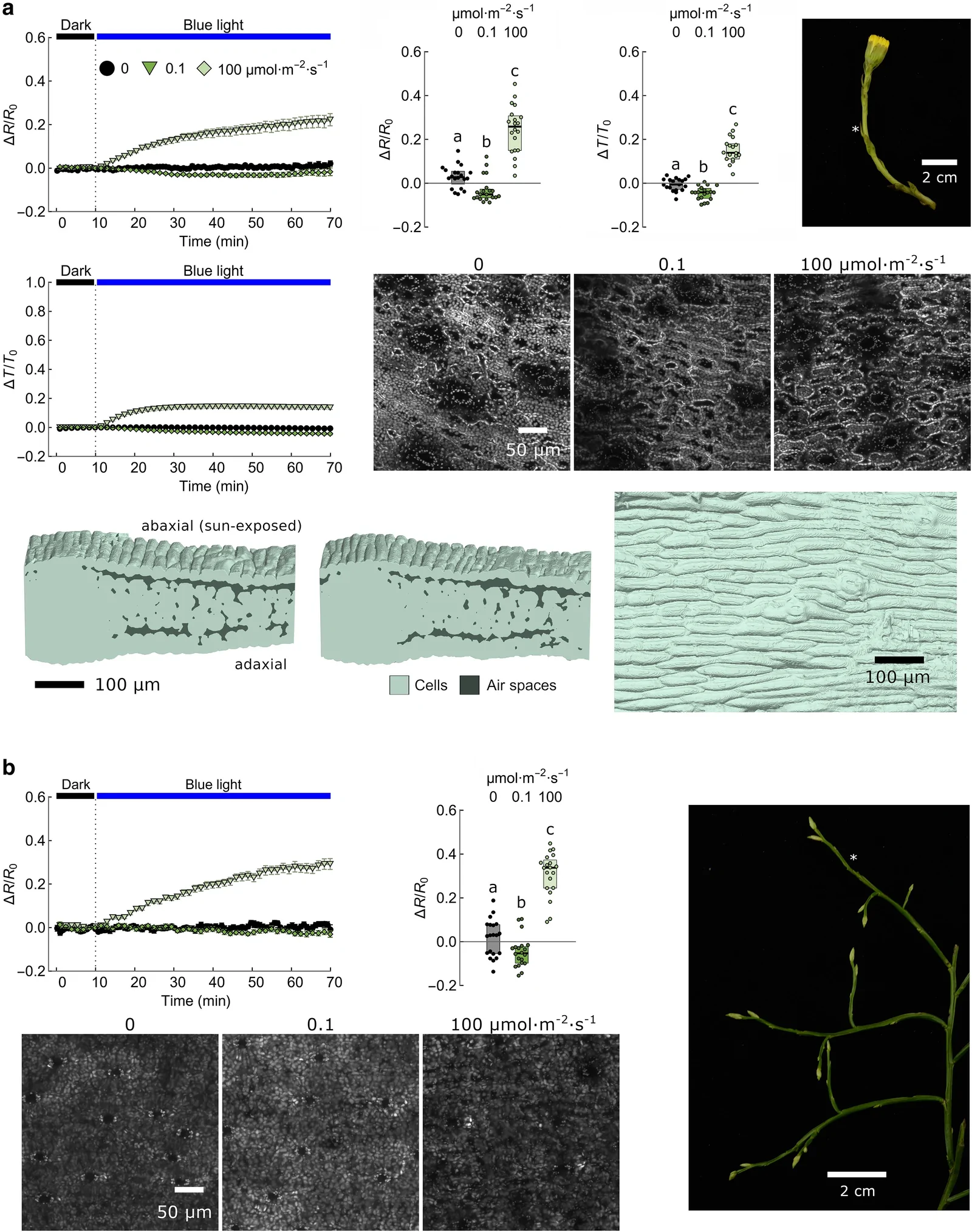
Detection of chloroplast movements by cross-polarized reflectance changes in green scales and stems. Chloroplast movements were examined through measurements of reflectance and transmittance, as well as microscopic observations, for *Tussilago farfara* a) and *Vaccinium myrtillus* b). Time courses show changes in red-light reflectance Δ*R* induced by 0.1 or 100 µmol·m^−2^·s^−1^ of blue light or mock irradiation of the sun-exposed (abaxial) *Tussilago* scales or *Vaccinium* stems, normalized by the reflectance $R_{0}$ of the sample in a dark-adapted state. The angle of incidence of beams was 55° while the angle of observation was 0° (diffuse reflectance recorded). The incident red light was *P*-polarized, while the *S*-polarized component of reflected light was measured. Changes in hemispherical transmittance Δ*T* from photometric measurements are shown for reference. Confocal microscopy images of *Tussilago* scales and *Vaccinium* stems were recorded after 1 h irradiation with blue light of 0.1 or 100 µmol·m^−2^·s^−1^, or mock irradiation. Chlorophyll autofluorescence was detected. Photographs show the developmental stage of plants at the time of the measurement, with the examined organs marked with asterisks. The X-ray microtomography images for *Tussilago farfara* a) show the scale cross-section and its sun-exposed surface. In box plots, the means of transformed values from groups that do not share any letter differ at the 0.05 level (Tukey's method, adjusted for multiple comparisons).

To link the changes in optical properties with the structure of the leaf, we investigated cross-sections of leaves with confocal microscopy (Figure S20) and larger leaf fragments with X-ray microtomography (Figs 5 and 6). The leaves of *D. verna* were ca. 200 to 250 µm thick, consisting of 2 to 3 layers of palisade mesophyll and 3 to 4 layers of spongy mesophyll (Figure S20a, Fig. 5a). The upper epidermis was covered with trichomes similar in shape to those of *Arabidopsis* (Fig. 5a). The leaves of *S. media* consisted of only 1 to 2 layers of palisade mesophyll, characteristic of species growing in shaded environments (Figure S20b, Fig. 5b). The intercellular spaces occupied a high fraction of the leaf. The epidermis comprised pavement cells with lobes and indentations (Fig. 5b). Epidermal cells of *D. verna* and *S. media* did not show any preferred direction in their shape or orientation. By contrast, the thin, monofacial scales of *T. farfara* were covered with epidermal cells elongated along a single axis (Figure S20c, Fig. 6a). Thus, any rotation of the scale around the normal to the blade may affect specular reflection from its surface. None of these species showed distinct epicuticular wax structures resolvable by optical microscopy, which could contribute to the surface Mie scattering. *V. myrtillus* stem was rectangular on its cross-section, with clear corners. It consisted of 2 types of cells containing chloroplasts (Figure S20d). There were 2 to 3 layers of small cells rich in chloroplasts just under the epidermis. The inner part of the stem was filled with larger cells that formed loose parenchyma (Figure S20d).

## Discussion

We show that changes in leaf directional reflectance for the most part mimic the changes in hemispherical transmittance, which is typically used for chloroplast movement detection, in agreement with previous reports (Seybold 1956; Zurzycki 1961; Dutta et al. 2015). Using a set of chloroplast movement mutants of *Arabidopsis thaliana*, we demonstrate that the observed changes in reflectance stem directly from different positions of the chloroplasts, not from other light-dependent processes in the leaf (Fig. 2). In the examined wild plant species, the relative sensitivity of reflectance-based detection was close to the sensitivity of the transmittance-based method. This suggests that remote monitoring of chloroplast movements in field conditions in response to changes in sunlight irradiance is feasible (Figs 5 and 6). The most pronounced difference between transmittance and reflectance changes was in the kinetics. Changes in the cross-polarized reflectance of the adaxial side of the leaf were slower than changes in transmittance. We observed this phenomenon in *Arabidopsis thaliana* (Figure S18), *Draba verna*, and, to a lesser extent, in *Stellaria media* (Fig. 5). The cause of this effect remains to be determined. However, it seems likely that reflectance changes are disproportionately affected by chloroplast movements in the mesophyll layer proximal to the measured leaf side, while transmittance changes are shaped more uniformly by movements across all layers of the mesophyll. We observed that the rate of cross-polarized reflectance changes is greater for the abaxial than adaxial side. This suggests that chloroplasts movements are faster in the spongy parenchyma than in the palisade

Our results indicate that chloroplast movements affect the diffuse component of reflectance (Fig. 2), while its specular component does not carry any information about chloroplast positioning. This is in line with the fact that specular reflection is not affected by the pigment composition of the leaf, as shown in studies on variegated leaves (Grant et al. 1987). Specular reflection arises mainly from the interaction between incoming radiation and the waxes or the cuticle. The prominence of specular lobes of the bidirectional reflectance distribution function depends on the angle of incidence and the roughness of the leaf surface (Breece and Holmes 1971; Bousquet et al. 2005). In our experiments, the presence of trichomes does not exert a considerable effect on light reflected from the leaves of *Arabidopsis thaliana* (hairy WT Col-0 versus bare *glabra1*) (Fig. 2). This is consistent with previous results obtained for the *glabra1* mutant, showing no significant difference in specular reflectance between this hairless line and lines bearing trichomes (Neuwirthová et al. 2021).

Strong dependence of detected leaf reflectance on incidence and observation angles may hinder the detection procedure (Fig. 2). Leaf movements (*eg*, nyctinastic or due to wind) increase the variance of the reflectance signal. Such deviations should average out when enough measurements are taken. Systematic errors, however, can arise from light-induced reorientation of leaves and shoots, either photonastic, phototropic, or wilting. The light-induced changes in leaf orientation have been observed in several plant families. They can be fast and substantial in magnitude. In *Oxalis oregana*, high blue light induces leaflet folding, with the petiole angle decreasing by 90° within a few minutes (Björkman and Powles 1981). In *Arabidopsis*, the blade of a leaf changes its orientation within a few hours in response to low blue light (Inoue et al. 2008). Such a rotation of the blade would change the angle of incidence of measuring light, affecting the amount of reflected light received by the detector. The dependence of reflectance of an *Arabidopsis* leaf on the observation angle is pronounced (Fig. 2), even though leaves in this species are not glossy. This indicates that a considerable magnitude of the spurious signal can be expected if chloroplast movements are examined through direct reflectance measurements of leaves that move, *eg*, in plants exposed to wind.

Here, we show that such issues can be successfully mitigated through polarization-sensitive detection (Fig. 3). The proposed approach improves the relative sensitivity of detection and sidesteps the undesirable angular dependence of leaf reflectance. The results obtained for wild species show that the cross-polarized reflectance can be used to investigate chloroplast movements in species that vary largely in morphology and architecture of photosynthetic organs (Figs 5 and 6). It is a convenient method for detecting chloroplast avoidance in photosynthetic stem tissues (Fig. 6b), which cannot be probed by the light transmittance method. The results obtained with the proposed method suggest that chloroplast movements in green stems of *V. myrtillus* may play a role in winter acclimation. At low temperatures, the sensitivity of chloroplast movements to light increases (Łabuz et al. 2015). Stems of *V. myrtillus* are equipped with stomata and photosynthetic tissue located directly beneath the epidermis, a feature shared with several genera of shrubs inhabiting extreme environments (Nilsen 1995; Ávila et al. 2014). Our findings provide additional background for understanding how the stems of *V. myrtillus* can carry out photosynthesis even in winter and early spring when the shoots are leafless (Havas 1971; Skre 1975; Stewart and Bannister 1973).

We have found that the increase in the leaf’s ability to depolarize light upon induction of the chloroplast avoidance response is correlated with the increase in reflectance (Fig. 4). Depolarization of light typically requires multiple random events (Rvachev and Guminetskii 1966; Egan et al. 1968; Code and Whitney 1995; Dogariu et al. 1997). The probability that light undergoes multiple scattering events without being absorbed decreases rapidly with the probability of absorption in the medium. This leads to an inverse relationship between albedo and the medium's ability to depolarize light (Cochenour et al. 2010). Thus, the magnitude of depolarization can be used to sense processes that change the reflectance of the sample. The relationship between reflectance and the degree of polarization of reflected light is often used to study astronomic objects, in particular through Umov’s relations (Sterzik et al. 2012; Shkuratov et al. 2015). The magnitude of depolarization usually exhibits only modest dependence on the orientation of the sample with respect to the incident beam and the angle of observation (Egan et al. 1968). Instead, it is dependent mostly on the phase angle, which is the angle between the incident beam and the direction of observation. This angle is directly controlled by the experimenter and can be fixed by the construction of the measuring apparatus. Despite its advantages in the remote sensing of samples whose geometry is beyond the experimenter's control (Krafft et al. 2024), polarimetry still receives relatively little attention as a tool for vegetation trait studies. The relationship between reflectance and depolarization appears to be exhibited also by leaves. For example, the leaf's ability to depolarize red laser light increases upon drying (Egan et al. 1968). It is greater for green light than for either blue or red light (Rvachev and Guminetskii 1966). Leaf veins depolarize light to a greater extent than the surrounding mesophyll tissue (Patty et al. 2018; Van Eeckhout et al. 2019). Depolarization of light reflected from leaves of 3 tree species is negatively correlated with their chlorophyll content (Yao et al. 2020).

The advantages of remote sensing of chloroplast movements would be two-fold. The first advantage is practical, as chloroplast movements influence not only the total reflectance of the leaf but also the shape of the spectrum of reflected light. As a result, they may affect the values of vegetation indices used in remote sensing, in particular, indices related to the pigment content (Hermanowicz and Łabuz 2025). Thus, a reliable method for detection of chloroplast positioning in conditions in which the leaf orientation or canopy geometry cannot be controlled may allow for the reduction of a confounding factor in the reflectance-based detection of other plant traits. In addition, the reflectance-based detection appears to be the only method that can achieve the throughput necessary to investigate the ecological role of chloroplast movements in ecosystems. The variability of chloroplast movements between different species is high (Königer and Bollinger 2012). Moreover, they exhibit substantial phenotypic plasticity, with their magnitude dependent on the light conditions during growth (Trojan and Gabrys 1996). The substantial effect of chloroplast positioning on the polarimetric properties of the leaf points to the possibility of using polarimetric remote sensing, for example, LiDAR-based Mueller matrix polarimetry (Keyser et al. 2019), or mast-mounted polarimeters (Krafft et al. 2024) for chloroplast movement detection in the field.

## Conclusions

The effect of chloroplast movements on directional leaf reflectance is substantial. However, changes in the signal due to leaf reorientation may be mistaken for chloroplast movements. In this work, we demonstrated for the first time that measurements of the cross-polarized reflectance and DoLP of reflected light alleviate this problem, enabling sensitive noncontact detection of chloroplast movements. We found that the chloroplast avoidance response increases leaf's ability to depolarize linearly polarized light, while the chloroplast accumulation response has the opposite effect. This indicates that the positioning of chloroplasts in the cell substantially affects the chance of light undergoing multiple scattering in the mesophyll. The developed methodology may be combined with already available hardware for remote sensing to improve the evaluation of physiological traits in the field.

## Supplementary Material

kiag739_Supplementary_Data

## Data Availability

The data that support the findings of this study are openly available in FigShare at https://doi.org/10.6084/m9.figshare.21082654 (reflectance and transmittance recordings for *Arabidopsis* WT and mutants) and https://doi.org/10.6084/m9.figshare.24424843 (wild plants). Java source code for the software is publicly available via GitHub at https://github.com/pawelHerm/beamJ/tree/master/BeamJ. Wolfram Mathematica package for ray tracing is available at https://github.com/plantPhotobiologyLab/openRayTracer.
