# Supplementary material for: Sensitive detection of chloroplast movements through changes in leaf cross-polarized reflectance
Source: Plant Physiol. 2026 Oct 6;202(2):kiag739. doi: 10.1093/plphys/kiag739 (PMC13714551; doi:10.1093/plphys/kiag739)
Supplement: Supplementary file 1 [file kiag739_supplementary_data.zip › Hermanowicz_Supplement_31_08_2026.docx]

**Sensitive detection of chloroplast movements through changes in leaf cross-polarized reflectance**

Paweł Hermanowicz, Aleksandra Giza, Katarzyna M. Sowa, Paweł Korecki, Justyna Łabuz

**Supplementary figures**

**Fig. S1. The reflectometric set-up used in the study.** (**A)**. Photo of the set-up. The rotating board provides control over the polar angle at which reflected light is detected. (**B)**. A model of the 3D printed sample holder used to mount the leaf in the gimbal. The holder features a hole to prevent reflection from the material beneath the leaf. **C**. The relationship between the blue and red beam relative irradiances and the distance from the beam centre.

**Fig. S2.** **Relative spectral irradiance as a function of wavelength** of the (**A**) actinic (measured maximum at 446 nm, FWHM 15 nm) and (**B**) measuring (660 nm, FWHM 9 nm) beams used in the reflectometric setup. The spectra were recorded with the Black Comet SR spectrometer (Stellar-Net). The cosine corrector of the probe was positioned in the leaf sample mount and oriented perpendicularly to the beams.

**Fig. S3.** **Red-light bidirectional reflectance of the adaxial side of dark-adapted *Arabidopsis* mutant leaves. *glabra1*, *phot1*, *phot2*, *phot1phot2,* and *jac1* *Arabidopsis* plants**, measured for the incidence angle of either 40° (**A**) or 55° (**B**) and several angles of observation. The incident beam was unpolarized. The transmission axis of the detector polarizer was either parallel (*P,* black dots) or perpendicular (*S*, yellow triangles) to the plane of incidence. Five leaves from different plants were examined for each combination of plant line and angle of incidence. Error bars show the standard error.

**Fig. S4. Changes in leaf reflectance due to chloroplast movements.** The amplitudes of red-light bidirectional reflectance changes induced by 1 h irradiation with blue light of 0.1 (**A**, **B**) or 100 µmol·m^-2^·s^-1^ (**C**, **D**) in leaves of dark-adapted *Arabidopsis thaliana* plants: wild-type (WT), *glabra1* and chloroplast movement mutants (*phot1*, *phot2*, *phot1phot2*, *jac1*). The blue actinic and red measuring beams were unpolarized and impinged on the adaxial leaf surface at an angle of 40°. For measurements of diffuse directional reflectance (**A**, **C**), the detector was positioned at 0° to the leaf's normal. For measurements of combined specular and diffuse directional reflectance, the detector was positioned at --40° (**B**, **D**). Two orientations of the detector polarizer were used: its transmission axis was either parallel to the plane of incidence (*P*, attenuates specular reflection, grey boxes) or perpendicular (*S*, yellow boxes). Boxes that do not share any letter represent groups for which the means of transformed values differ at the 0.05 level (Tukey's method, adjusted for multiple comparisons).

 **Fig. S5.** **Changes in red-light reflectance due to low blue light at 40° incidence.** Time courses of changes in red-light bidirectional reflectance induced by 1 h irradiation with low blue light (0.1 µmol·m^-2^·s^-1^) of the adaxial surface of *Arabidopsis* leaves. As marked in the figure, each row of the matrix of charts corresponds to a different plant line, from the top: wild type (WT), *glabra1, phot1*, *phot2*, *phot1phot2,* and *jac1* mutants. The angle of incidence of the measuring and actinic beams was 40°. The incident beams were not polarized. The detector was positioned at an angle of 0° (the detector normal parallel to the leaf normal) or -40°. The transmission axis of the detector polarizer (analyser) was either parallel (*P,* black dots) or perpendicular (*S*, yellow triangles) to the plane of incidence. The charts in the first two columns show raw reflectance recordings *R*, while the curves in the third and fourth columns show the difference between current reflectance and the reflectance at the onset of blue light irradiation *ΔR*. Error bars show the standard error, the plot markers (black dots for the *P* polarizer orientation, yellow triangles for the *S* orientation) show mean values.

**Fig. S6.** **Changes in red-light reflectance due to low blue light at 55° incidence**. Time courses of changes in red-light reflectance induced by 1 h irradiation with low blue light (0.1 µmol·m^-2^·s^-1^) of the adaxial surface of *Arabidopsis* leaves. As marked in the figure, each row of the matrix of charts corresponds to a different plant line, from the top: wild type (WT), *glabra1, phot1*, *phot2*, *phot1phot2,* and *jac1* mutants. The angle of incidence of the measuring and actinic beams was 55°. The incident beams were not polarized. The observation angle was 0° (diffusely reflected light recorded) or -55° (both diffusely and specularly reflected light recorded). The transmission axis of the detector polarizer (analyser) was either parallel (*P,* black dots) or perpendicular (*S*, yellow triangles) to the plane of incidence. The charts in the first two columns show raw bidirectional reflectance recordings *R*, while the curves in the third and fourth columns show the difference *ΔR* between current reflectance and the reflectance at the onset of blue light irradiation. Error bars show the standard error. The plot markers (black dots for the *P* polarizer orientation, and yellow triangles for the *S* orientation) show mean values.

**Fig. S7. Changes in leaf transmittance due to chloroplast movements.** Time courses of changes in red-light hemispherical transmittance, induced by blue light of 0.1 (**A**) or 100 µmol·m^-2^·s^-1^ (**B**) in rosette leaves of 5-week-old *Arabidopsis* plants. 17 leaves from different individual plants were measured for each combination of plant line and irradiance level. Error bars show the standard error. Amplitudes (**C**) and maximal rates (**D**) of changes in transmittance are calculated from curves in (**A**) and (**B**). The significance of differences between groups is assessed with Tukey’s test on asinh-transformed values. The means of transformed values from groups that do not share a letter are different at the 0.05 level (adjusted for multiple comparisons).

 **Fig. S8.** **Changes in red-light reflectance due to high blue light at 40° incidence.** Time courses of changes in red-light reflectance induced by 1 h irradiation with high blue light (100 µmol·m^-2^·s^-1^) of the adaxial surface of *Arabidopsis* leaves. As marked in the figure, each row of the matrix of charts corresponds to a different plant line, from the top: wild type (WT), *glabra1, phot1*, *phot2*, *phot1phot2,* and *jac1* mutants. The angle of incidence of the measuring and actinic beams was 40°. The incident beams were not polarized. The observation angle was 0° (diffusely reflected light recorded) or -40° (both diffusely and specularly reflected light recorded). The transmission axis of the detector polarizer (analyser) was either parallel (*P,* black dots) or perpendicular (*S*, yellow triangles) to the plane of incidence. The charts in the first two columns show raw reflectance recordings *R*, while the curves in the third and fourth columns show the difference between current reflectance and the reflectance at the onset of blue light irradiation *ΔR*. Error bars show the standard error, the plot markers (black dots for the *P* polarizer orientation, yellow triangles for the *S* orientation) show mean values.

**Fig. S9.** **Changes in red-light reflectance due to high blue light at 55° incidence.** Time courses of changes in red-light reflectance induced by 1 h irradiation with high blue light (100 µmol·m^-2^·s^-1^) of the adaxial surface of *Arabidopsis* leaves. As marked in the figure, each row of the matrix of charts corresponds to a different plant line, from the top: wild type (WT), *glabra1, phot1*, *phot2*, *phot1phot2,* and *jac1* mutants. The angle of incidence of the measuring and actinic beams was 55°. The incident beams were not polarized. The observation angle was 0° (diffusely reflected light recorded) or -55° (both diffusely and specularly reflected light recorded). The transmission axis of the detector polarizer (analyser) was either parallel (*P,* black dots) or perpendicular (*S*, yellow triangles) to the plane of incidence. The charts in the first two columns show raw reflectance recordings *R*, while the curves in the third and fourth columns show the difference between current reflectance and the reflectance at the onset of blue light irradiation *ΔR*. Error bars show the standard error. The plot markers (black dots for the *P* polarizer orientation, and yellow triangles for the *S* orientation) show mean values.

**Fig. S10.** **Rates of red-light reflectance changes due to high blue light.** The maximal rates of red-light reflectance changes induced by 1 h irradiation with blue light of 0.1 (**A**, **B**, **E**, **F**) or 100 µmol·m^-2^·s^-1^ (**C**, **D**, **G**, **H**) were calculated for leaves of dark-adapted *Arabidopsis* plants: wild type (WT), *glabra1* and chloroplast movement mutants (*phot1*, *phot2*, *phot1phot2*, *jac1*). The blue actinic and red measuring beams were unpolarized and impinged on the adaxial leaf surface at the angle of 40° (**A**-**D**) or 55° (**E**-**H**). For measurements of diffuse directional reflectance (**A**, **C**, **E**, **G**), the observation angle was 0°. For measurements of combined specular and diffuse directional reflectance, the detector was positioned at -40° (**B**, **D**) or -55° (**F**, **H**). For each observation angle, two orientations of the detector polarizer (analyser) were used: its transmission axis was either parallel to the plane of incidence (*P*, attenuates specular reflection, grey boxes) or perpendicular (*S*, yellow boxes). Boxes that do not share any letter represent groups for which the means of transformed values differ at the 0.05 level (Tukey's method, adjusted for multiple comparisons).

**Fig. S11. Red-light reflectance of dark-adapted leaves.** Red light reflectance of the adaxial side of leaves detached from dark-adapted *Arabidopsis* wild type (WT), *glabra1,* and chloroplast movement mutants (*phot1*, *phot2*, *phot1phot2*, *jac1*). The angle of incidence of unpolarized measuring light was either 40° (**A**, **B**) or 55° (**C**, **D**), while the angle of observation was set to 0° (diffuse reflectance recorded) (**A**, **C**) or either -40° (**B**) or -55° (**D**) (combined specular and diffuse reflectance recorded). The transmission axis of the analyser was either parallel (*P,* grey boxes) or perpendicular (*S*, yellow boxes) to the plane of incidence. Groups that do not share any letter differ at the 0.05 level in means of transformed values (Tukey's method, adjusted for multiple comparisons).

**Fig. S12**. **The sensitivity of red-light leaf reflectance to the chloroplast positioning**. The sensitivity was calculated as the ratio *ΔR/R_0_* between reflectance change *ΔR* induced with blue light of 0.1 (**A**, **B**, **E**, **F**) or 100 µmol·m^-2^·s^-1^ (**C**, **D**, **G**, **H**) and the initial reflectance *R_0_* of the dark-adapted leaf. The angle of incidence of actinic and measuring beams at the adaxial leaf surface was 40° (**A** - **D**) or 55° (**E** - **H**). The incident beams were not polarized. To measure diffuse reflectance (**A**, **C**, **E**, **G**), the observation angle was 0°. To measure combined specular and diffuse reflection (**B**, **D**, **F**, **H**), the observation angle was -40° or -55°. For observation angle, two analyser orientations were used: its transmission axis was either parallel to the plane of incidence (*P*, attenuates specular reflection, grey boxes) or perpendicular (*S*, yellow boxes). Boxes that do not share any letter represent groups for which the means of transformed values differ at the 0.05 level (Tukey’s method, adjusted for multiple comparisons).

**Fig. S13.** **Amplitudes and rates of changes in the polarized red-light reflectance** **due to chloroplast movements**. The adaxial surface of leaves of wild-type (WT) and *phot1phot2* *Arabidopsis* plants was irradiated for 1 h with high blue light (100 µmol·m^-2^·s^-1^). The incidence angle of actinic and measuring beams was 55°. The amplitudes (**A**, **B**) and maximal rates of reflectance changes (**C**, **D**) were calculated. For measurements of diffuse reflectance (**A**, **C**), the observation angle was 0°. For measurements of combined specular and diffuse reflectance (**B**, **D**), the observation angle was -55°. Four possible combinations of the orientation (*P/S*) of the polarizers in front of the measuring light source and the detector were examined. Boxes that do not share any letter represent groups for which the means of transformed values differ at the 0.05 level (Tukey's method, adjusted for multiple comparisons).

**Fig. S14**. **Reflectance time courses used to examine the degree of linear polarization of light**. Changes in the polarized red-light reflectance induced by 1 h irradiation with blue light of 0.1 (**A**-**D**) or 100 µmol·m^-2^·s^-1^ (**E**-**H**) of the abaxial or adaxial surface of leaves of wild type (WT) and *phot1phot2* *Arabidopsis* plants. The axis of the analyzer in front of the detector was rotated during the experiment from the *S* to *P* position. The incidence angle of actinic and measuring beams was 55°. The observation angle was 0°.

**Fig. S15**. **Effect of chloroplast movements on the leaf’s ability to depolarize light**. The red measuring light was *P*-polarized, incident at 55° on the adaxial side, and the observation angle was -55°. (**A**) The degree of linear polarization (DoLP) of light reflected from dark-adapted wild-type and *phot1phot2* *Arabidopsis* leaves. (**B** - **C**) Changes in the degree of linear polarization of reflected light (**B**) and total reflectance (**C**) due to irradiation with blue light. (**D** - **E**) The relationship between the total reflectance and the degree of polarization of the red beam reflected from dark-adapted or high-blue-light irradiated leaves, for the wild -type (**D**) and *phot1phot2* mutant (**E**). (**A - C**) Boxes that do not share any letter represent groups for which the means of transformed values differ at the 0.05 level (Tukey's method, adjusted for multiple comparisons).

**Fig. S16**. **Control measurements for experiments on the effect of chloroplast movements on the leaf’s ability to depolarize light.** (**A**) The degree of linear polarization (DoLP) of light reflected from dark-adapted wild-type and *phot1phot2* *Arabidopsis* leaves. The red measuring light was *P*-polarized, incident at 55°, and the observation angle was 0°. (**B**) Total chlorophyll content of *Arabidopsis* WT and *phot1phot2* mutant leaves. (**C** - **D**) Amplitudes of changes in red-light transmittance induced by 1 h irradiation with continuous blue light of 0.1 or 100 µmol·m^-2^·s^-1^, incident at the abaxial or adaxial side of wild-type *Arabidopsis* leaves. Boxes that do not share any letter represent groups for which the means of transformed values differ at the 0.05 level (Tukey's method, adjusted for multiple comparisons).

**Fig. S17.** **Anatomy of Arabidopsis leaves used in the study**. (**A**) Cross-sections of *Arabidopsis* WT and *phot1phot2* mutant leaves imaged with confocal microscopy, using chlorophyll autofluorescence (upper row) and transmitted laser light (lower row). (**B**) The adaxial surface and the mesophyll of wild-type leaves examined with X-ray microtomography.

**Fig. S18.** **Chloroplast movements examined with reflectometric, photometric, and microscopic observations in wild-type *Arabidopsis***. Time courses and amplitudes of changes in red-light reflectance **(A, B)** and hemispherical transmittance **(C, D)** induced by 1 h irradiation with low (0.1) or high blue light (100 µmol·m^-2^·s^-1^) or mock irradiation of the adaxial side of leaves. The angle of incidence of beams was 55°, while the angle of observation was 0° (diffuse reflectance recorded). The incident measuring light was polarized in the direction parallel to the plane of incidence (*P*). The polarizer in front of the detector (analyzer) transmitted light perpendicular to the incidence plane (*S*). The means of transformed values from groups that do not share any letter differ at the 0.05 level (Tukey's method, adjusted for multiple comparisons). Confocal microscopy images **(E)** of leaf tissue after 1 h irradiation with low blue light (0.1 µmol·m^-2^·s^-1^) or high blue light (100 µmol·m^-2^·s^-1^) or mock irradiated.

**Fig. S19.** **Effect of the leaf side on reflectance changes induced by high blue light in *Arabidopsis***. (**A** – **B**) Amplitudes (**A**) and rates (**B**) of changes in leaf cross-polarized reflectance for measuring red light, incident at either the adaxial or abaxial side of wild-type and *phot1phot2* leaves. The changes were induced by 1 h irradiation with blue light of 100 µmol·m^-2^·s^-1^. (**C)**. Cross-polarized reflectance of dark-adapted leaves. (**D)**. Traces of changes in cross-polarized reflectance induced by blue-light irradiation. In **A** – **D**, the angle of incidence of beams was 55°, while the angle of observation was 0° (diffuse reflectance recorded). The incident measuring light was polarized in the direction parallel to the plane of incidence (*P*). The polarizer in front of the detector (analyzer) transmitted light perpendicular to the incidence plane (*S*). In **A** – **C**, means of transformed values from groups that do not share any letter differ at the 0.05 level (Tukey's method, adjusted for multiple comparisons).

**Fig. S20. Anatomy of the photosynthetic structures of wild plants used in the study.** Laser scanning confocal microscopy images of cross-sections of *Draba verna* (**A**)*, Stellaria media* (**B**) leaves*, Tussilago farfara* (**C**) flowering shoot scales, and the *Vaccinium myrtillus* (**D**) stem. Chlorophyll autofluorescence (in A - D) and transmitted light (in A - C) images are shown. Scale bars 100 µm.
