# Supplementary material for: Sensitive detection of chloroplast movements through changes in leaf cross-polarized reflectance
Source: Plant Physiol. 2026 Oct 6;202(2):kiag739. doi: 10.1093/plphys/kiag739 (PMC13714551; doi:10.1093/plphys/kiag739)
Supplement: Supplementary file 1 [file kiag739_supplementary_data.zip › Supporting_Materials_1.docx]

**Sensitive detection of chloroplast movements through changes in leaf cross-polarized reflectance**

Paweł Hermanowicz, Aleksandra Giza, Katarzyna Sowa, Paweł Korecki, Justyna Łabuz

*Optomechanical setup for measurement of blue light-induced changes in leaf bidirectional reflectance*

The scheme of the setup is shown in Fig. 1 A and Fig. S1. The measuring beam was produced by a red LED (nominal wavelength of 660 nm, manufacturer part number M660L4, Thorlabs) and collimated with an achromatic lens (#49778, Edmund Optics). The collimated beam was passed through a bandpass interference filter (660 nm max, 10 nm FWHM, #15-126, Edmund Optics) to reduce the width of its spectrum. In some experiments (Fig. 1 C), a linear polarizer (40 dB extinction ratio, #47316, Edmund Optics) was inserted next to the filter. The actinic beam was supplied by a blue LED (nominal wavelength of 455 nm, M455L4, Thorlabs), collimated with an aspherized achromatic lens (#49-662, Edmund Optics), and passed through a bandpass interference filter (450 nm, 25 nm FWHM, #15146, Edmund Optics). The spectra of the beams measured at the leaf mount are shown in Fig. S2. The beams were combined with a dichroic mirror (DMLP550, Thorlabs) and passed through a condenser composed of two achromatic lenses (AC 254-040-A, Thorlabs) and an iris diaphragm. The beam uniformity was examined with a CDD camera (Axiocam MRm rev3, Carl Zeiss, Jena). The standard deviation of irradiance of the blue beam within the area probed with red light was equal to ca. 1% of the average irradiance (Fig. S1 C). The size of the beams was controlled with field diaphragms. The radius of the red beam at the sample plane was set to 1.5 mm, to avoid illuminating the midvein, while its power was ca. 29 µW (when unpolarized) or ca. 13 µW (when polarized). The radius of the blue beam was 6 mm. The field of view of the detector (ca. 12 mm in radius) exceeded the size of the red spot. Thus, the detected signal was proportional to radiant intensity and was divided by the cosine of the observation angle to obtain radiance. The leaf was placed in a custom-made holder (Fig. S1 B) mounted in a gimbal (GMB1, Thorlabs), which allowed for control of the angle between the normal to the leaf surface and the direction of incident light. The holder consisted of two parts, both with an elliptical hole in the middle, between which the leaf fragment was inserted. The edges of the specimen were covered with wet tissue paper. The angle between the incident beam and the leaf normal was kept at 40° or 55°. In our experiments, the reflected light was collected at the angle of observation of 0° (diffuse reflection) and either -40° or -55° (diffuse and specular reflection) with respect to the leaf normal. It passed through a linear polarizer (#47316, Edmund Optics) and a bandpass interference filter (660 nm, #15-126, Edmund Optics), blocking actinic light and most of the chlorophyll fluorescence. The filtered light was focused on a photodetector (amplified silicon photodiode, PDA100A2, Thorlabs) with a plano-convex lens (LA1074-A, Thorlabs). Using an integrating sphere (2P4/M, Thorlabs) to obtain a polarization-insensitive reference detector (McClain et al., 1995), we found that the sensitivity of this photodiode varied by 0.1% with the polarization plane orientation. The angular size of the clear aperture of the collecting lens with respect to the sample center was 0.019 steradian (calculated using our ray-tracing Mathematica package <https://github.com/plantPhotobiologyLab/openRayTracer>). To control the observation angle, the detector was mounted on the rotation stage (RBB300A/M, Thorlabs). The experiments were performed with a polarizer in front of the red LED, either present (Fig. 1 C) or removed (Fig. 1 B). When it was removed, the degree of linear polarization of the measuring beam at the sample plane was ca. 0.02. We refer to this as unpolarized light. The second polarizer, i.e., the analyser in front of the detector, was always installed. Its transmission axis was either set to parallel (transmits *P*) or perpendicular (transmits *S* component) to the plane of incidence. The LEDs supplying the measuring and the actinic beams were controlled with two programmable current sources (Cyclops drivers, (Newman et al., 2015)) that enabled precise regulation of light power. The measuring beam was amplitude-modulated at 1013 Hz using a sinusoidal signal generated with a field-programmable gate array (FPGA) board (STEMLab 125-14, Red Pitaya, Slovenia). The signal from the detector was fed into the FPGA board, running a lock-in amplifier program (Luda et al., 2019). Modulation of the amplitude of the measuring beam, in combination with the lock-in amplification (detection of a single frequency), improves the signal-to-noise ratio. In our setup, the additional benefit of lock-in amplification was a further reduction of the risk of detection of reflected unmodulated blue light. The actinic beam was controlled from a PC running BeamJ, our publicly available Java application (<https://github.com/pawelHerm/beamJ>). The application was designed to control custom optomechanical systems for the analysis of light-induced changes in the optical properties of the leaves. A silver mirror (#49194, Edmund Optics) placed in the gimbal was used to record the detected signal produced when the radiant flux incident on the sample was completely reflected. A white, diffusely reflecting polytetrafluoroethylene (PTFE) (Weidner and Hsia, 1981) disk (SM05CP2C, Thorlabs) was also measured whenever the setup was calibrated. However, the reported values of bidirectional reflectance are absolute (not divided by the PTFE signal). Axes of polarizers were determined using the two-polarizer method of (Rowell et al., 1969). The axis of the analyser was additionally checked using light that underwent *S*-polarization upon reflection from a glass window (#83375, Edmund Optics), placed in the sample gimbal, and illuminated with the measuring beam at Brewster’s angle. Leaf reflectance curves were processed with a custom-written Mathematica (Wolfram Research, USA) script.

**Reference list**

**Luda MA, Drechsler M, Schmiegelow CT, Codnia J**. **2019**. Compact embedded device for lock-in measurements and experiment active control. *Review of Scientific Instruments* **90**: 023106.

**McClain SC, Bartlett CL, Pezzaniti JL, Chipman RA**. **1995**. Depolarization measurements of an integrating sphere. *Applied Optics* **34**: 152–154.

**Newman JP, Fong MF, Millard DC, Whitmire CJ, Stanley GB, Potter SM**. **2015**. Optogenetic feedback control of neural activity. *eLife* **4**: e07192.

**Rowell RL, Levit AB, Aval GM**. **1969**. Determination of the Absolute Alignment of a Polarizer. *Applied Optics* **8**: 1734.

**Weidner VR, Hsia JJ**. **1981**. Reflection properties of pressed polytetrafluoroethylene powder. *Journal of the Optical Society of America* **71**: 856–861.
